# Charing Cross Hospital

**Published:** 1902-07-05

**Authors:** 


### CHARING CROSS HOSPITAL.

### LAYING OF FOUNDATION-STONE.

On June 20th the Duke of Connaught, as Grand Master of English Freemasons, laid the foundation-stone of the new building of the Charing Cross Hospital, with full Masonic ceremony, in the presence of Princess Louise, Duchess of Argyll, President of the hospital, and a large company.

At the hospital His Royal Highness was received by the Royal President and a deputation of grand officers.

Princess Louise, as President of the hospital, began the proceedings by requesting the Grand Master to lay the foundation-stone in Masonic form.

The Grand Master said he should be happy to comply with the request of Her Royal Highness, and, the upper stone having been raised and the vessels of corn, wine, and oil having been placed near at hand, the Duke called upon the Grand Chaplain to offer prayer, after which, at the bidding of the Grand Master, the stone-laying was proceeded with. It is an interesting fact that the mallet used by the Grand Master is the instrument with which Charles II. laid the foundation-stone of St. Paul's Cathedral. It was, after that ceremony, presented to to the Masonic body, and has for many years been in the care of the Lodge of Antiquity.

The Grand Master next scattered corn on the stone "as the emblem of plenty and abundance," saying "May the blessings of morality and virtue flourish within this building, producing fruit a hundredfold." He also poured out wine, "the emblem of joy and gladness," and sprinkled oil upon the stone, "the emblem of peace and unanimity," wishing prosperity, happiness, and good will amongst those who would assemble in the building.

Mr. Drummond moved a vote of thanks to his Royal Highness for attending, which was seconded by Sir Joseph Fayrer, and agreed to, the audience rising and giving three cheers.

The Grand Master, who was again loudly cheered, said: Your Royal Highness, my Lords, Ladies, and Brethren, I am indeed very sensible of the kind words which have fallen from Mr. Drummond and Sir Joseph Fayrer, and it requires no vote of thanks for me to assure you of the great pleasure with which I have attended here to-day in compliance with the wishes of my beloved sister, your president—(cheers)—to lay this foundation stone with full Masonic honours. I may speak in the name of Grand Lodge and all Masons, and say that we, a large and influential body of his Majesty's most loyal subjects, appreciate very much the opportunity of performing this ceremony, which I hope has interested those who have seen it perhaps for the first time, and which will, if possible, raise their opinion of Freemasons, and enable them to see what a good body of loyal Englishmen Freemasons are. (Cheers.) This hospital has special connection with the members of my family. My late lamented brother, the Duke of Edinburgh, was long president of it, and now my sister, Princess Louise, Duchess of Argyll, is your president. (Cheers.) I do hope and believe that under her presidency this hospital will flourish in the manner we would all wish it to do. Our beloved Sovereign the King has for many years taken a deep interest in everything connected with the hospitals of this metropolis—(cheers)—and he has used every endeavour to promote and increase the funds which are necessary for the working of these all-important but very expensive establishments. I am sure that this extension will largely increase the value of the institution and its results. We have heard of the large nursing home and other buildings recently completed, and I hope that when this new extension, the foundation stone of which I have had the great pleasure and honour of laying, is completed, it will make the Charing Cross Hospital one of the finest and most complete of that splendid number of hospitals of which we are all so proud in this city of London. (Cheers.) I thank you for the reception you have given me and for the kind vote of thanks which has been recorded.

At the request of the Treasurer, his Royal Highness named one of the new wards the Alfred ward, in memory of the Duke of Edinburgh; but, ascertaining that there was already an Alfred ward, the Duke said he felt sure he should be consulting the wishes of those present by naming it the Louise ward. (Cheers.)

The proceedings then terminated.

